# Efficacy and mechanism of acupuncture in animal models of depressive-like behaviors: a systematic review and meta-analysis

**DOI:** 10.3389/fnins.2024.1330594

**Published:** 2024-02-15

**Authors:** Yingjie Huang, Weiping Chen, Xingfu Li, Tian Tan, Tunyi Wang, Shishi Qiu, Guangyao Li, Cong Yang, Min Li, Lining Duan

**Affiliations:** ^1^The First Affiliated Hospital of Guangzhou University of Chinese Medicine, Guangzhou, China; ^2^Clinical Medical of Acupuncture, Moxibustion and Rehabilitation, Guangzhou University of Chinese Medicine, Guangzhou, China; ^3^The Second Clinical College of Guangzhou University of Chinese Medicine, Guangzhou University of Chinese Medicine, Guangzhou, China; ^4^Science and Technology Innovation Center, Guangzhou University of Chinese Medicine, Guangzhou, China

**Keywords:** acupuncture, depression, depressive-like behaviors, animal models, systematic review, meta-analysis

## Abstract

**Background:**

Many studies have investigated the efficacy of acupuncture in treating depression, but the mechanism of acupuncture for depression is still controversial and there is a lack of meta-analysis of mechanisms. Consequently, we investigated acupuncture’s efficacy and mechanism of depression.

**Methods:**

We searched the Cochrane Library, PubMed, EMBASE, Web of Science. The SYRCLE Risk of Bias Tool was used to assess bias risk. Meta-analysis was performed using Stata 15.0 for indicators of depression mechanisms, body weight and behavioral tests.

**Results:**

A total of 22 studies with 497 animals with depressive-like behaviors were included. Meta-analysis showed that acupuncture significantly increased BDNF [SMD = 2.40, 95% CI (1.33, 3.46); *I*^2^ = 86.6%], 5-HT [SMD = 2.28, 95% CI (1.08, 3.47); *I*^2^ = 87.7%] compared to the control group (*p* < 0.05), and significantly reduced IL-1β [SMD = −2.33, 95% CI (−3.43, −1.23); I2 = 69.6%], CORT [SMD = −2.81, 95% CI (−4.74, −0.87); *I*^2^ = 86.8%] (*p* < 0.05). Acupuncture improved body weight [SMD = 1.35, 95% CI (0.58, 2.11); *I*^2^ = 84.5%], forced swimming test [SMD = −1.89, 95% CI (−2.55, −1.24); *I*^2^ = 76.3%], open field test (crossing number [SMD = 3.08, 95% CI (1.98, 4.17); *I*^2^ = 86.7%], rearing number [SMD = 2.53, 95% CI (1.49, 3.57); *I*^2^ = 87.0%]) (*p* < 0.05) compared to the control group.

**Conclusion:**

Acupuncture may treat animals of depressive-like behaviors by regulating neurotrophic factors, neurotransmitters, inflammatory cytokines, neuroendocrine system.

**Systematic review registration:**

https://www.crd.york.ac.uk/prospero/display_record.php?ID=CRD42023403318, identifier (CRD42023403318).

## 1 Introduction

Depression is characterized by high prevalence, relapse rates, and disability as the most common mental health problem. More than 350 million people worldwide are affected by major depression (Uchida et al., 2018), and up to one third of patients have a lifetime prevalence of suicide attempts (Dong et al., 2019). With the rapid increase in prevalence, WHO predicted that depression will be number one on the global burden of disease list by 2030 (Malhi and Mann, 2018). Both the etiology and the pathophysiological mechanisms of depression remain unknown. There are several major academic hypotheses such as neurotransmitters, neurotrophic factors and neuropeptides such as brain-derived neurotrophic factor (BDNF), neurohormonalendocrine-related HPA axis, and inflammatory responsedominated by pro-inflammatory cytokines (Xu et al., 2021; Fries et al., 2023). Representative indicators of these mechanistic hypotheses (5-HT, BDNF, corticosterone, IL-1β) have been used by many research, the onset and treatment of depression is associated with these indicators. The main treatment for depression is psychotropic drug, and in the treatment of moderate to severe depression, antidepressants are the first line therapy (De Crescenzo et al., 2020). Although antidepressants are effective, their resistances are also evident (Marwaha et al., 2023). Antidepressants also have significant side effects, which can cause digestive adverse effects and loss of libido (Rothmore, 2020; Oliva et al., 2021), but also have the potential to cause mania or hypomania, and discontinuation of antidepressants is likely to result in “discontinuation syndrome” (Fava, 2014). Therefore, we urgently need an effective and non-toxic treatment that can be adhered to for a long time to solve the current treatment dilemma of depression.

Acupuncture had been used for thousands of years. In 2016, the American College of Physicians Clinical Practice Guidelines included acupuncture as a complementary alternative therapy for depression (Qaseem et al., 2016). Some high-quality clinical studies had demonstrated the significant efficacy of acupuncture for depression (Xu et al., 2023), and animal studies had also found that acupuncture improves behavioral tests in animal models of depressive-like behaviors (Zheng et al., 2019). To our knowledge, Kou et al. (2017) showed that acupuncture improved the behavior of depression in animals, but the quality of the included studies was not high, and the mechanisms of acupuncture were not demonstrated. Because only animal behavior was analyzed and pathophysiology was lacking. Also, there are no meta-analysis analyzing the acupuncture’s major mechanism indicators on depressive-like behaviors in animals.

Therefore, we conducted a comprehensive meta-analysis of animal experiments to explore the effects of acupuncture on relevant indicators (5-HT, BDNF, corticosterone, IL-1β) and efficacy (body weight, behavioral tests) in animals with depressive-like behaviors. In order to elucidate the efficacy and mechanism of acupuncture on depressive-like behaviors, and to provide guidance for clinical experiments, as well as to serve as a resource for clinical judgment and guideline development.

## 2 Methods

This study was registered in the PROSPERO International Systematic Review Prospective Register (CRD42023403318). This study followed the latest PRISMA guidelines for systematic reviews and meta-analyses^1^ (Page et al., 2021).

### 2.1 Search strategy

We searched PubMed, EMBASE, the Cochrane Library, and Web of science from database inception to January 2023. We used the following subject terms: “electroacupuncture,” “acupuncture,” “depression,” and “animals.” The detailed search strategy was presented in Appendix 1. Studies were restricted to English.

### 2.2 Inclusion criteria

The inclusion and exclusion criteria for this study were jointly developed by the two investigators (YJH and WPC). The inclusion criteria were as follows: ① randomized controlled trials of animals with depressive-like behaviors; ② successful establishment of depressive-like behaviors in animals with different modeling methods; ③ manual acupuncture or electroacupuncture in the test group; ④ depression animals without any intervention in the control group; ⑤ The study results included at least one indicator of depression mechanism (5-HT, BDNF, CORT, IL-1β).

### 2.3 Exclusion criteria

① Non-randomized or semi-randomized controlled trials; ② depressive-like behaviors caused by other diseases; ③ test groups were other acupuncture therapies or combined with other therapies; ④ studies did not include pre-determined outcomes; ⑤ systematic reviews or conference papers; ⑥ studies not in English.

### 2.4 Study screening and data extraction

Two researchers (YJH and WPC) independently screened the study, extracted the data and cross-checked them. Third party (XFL) were consulted or discussed in order to resolve disputes. During study screening, duplicate studies were first excluded, then titles were read, and after excluding apparently irrelevant studies, abstracts were further read, and finally the full text was read to determine if they were included. If needed, the original study authors were contacted by email and telephone to obtain information that had not yet been identified but was important to the study. We extracted the following data: ① first author’s name and publication year, ② basic characteristics of the animals with depressive-like behaviors, ③ method of modeling depression, ④ intervention characteristics, ⑤ outcomes (extracted depression-related mechanistic indicators as the primary outcome, the body weight and behavioral tests as secondary outcomes). The graphs were digitized with GetData Graph Digitizer 2.26 if only graphical data were provided.

### 2.5 Risk of study bias

The Systematic Review Center for Laboratory Animal Experiments (SYRCLE) Risk of Bias Tool (RoBT) was employed by two independent researchers (YJH, WPC), categorized as low risk, high risk, and unclear risk (Hooijmans et al., 2014). This RoBT assesses bias in the following 10 domains: ① sequence generation, ② baseline characteristics, ③ allocation concealment, ④ random housing, ⑤ caregiver and investigator blinding, ⑥ randomized outcome assessment, ⑦ outcome assessment blindness, ⑧ incomplete outcome data, ⑨ selective outcome reporting, ➉ other bias. Two investigators resolved assessment disputes through negotiation, and a third investigator (XFL) could be contacted for arbitration if necessary.

### 2.6 Statistics

Statistics 15.0 software was used for data analysis. The outcomes included were all continuous variables. A standardized mean difference (SMD) with its 95% confidence interval (CI) should be used when the results of included studies were reported in different measurements or units, a statistically significant difference was defined as *p*<0.05. We assessed heterogeneity by the *I*^2^ test. Fixed-effects model was used when *I*^2^ ≤ 50%, whereas random-effects model was used otherwise. We performed subgroup analyses for different acupuncture methods (EA MA), courses, modeling methods, strain to assess the effect of these factors on the meta results as well as heterogeneity. Egger’s test and the funnel plot were used to examine publishing bias, with sensitivity analyses completed to test the results’ stability and reliability.

### 2.7 Certainty assessment

The certainty of evidence for the primary outcome was assessed using GDT software according to the grading guidelines.^2^ It based on study design domain, risk of bias, inconsistency, indirectness, imprecision, and other considerations such as publication bias, effect size, and potential confounders. The quality of the final evidence was categorized as high, moderate, low, and very low.

## 3 Results

### 3.1 Study search result

**Figure 1:**
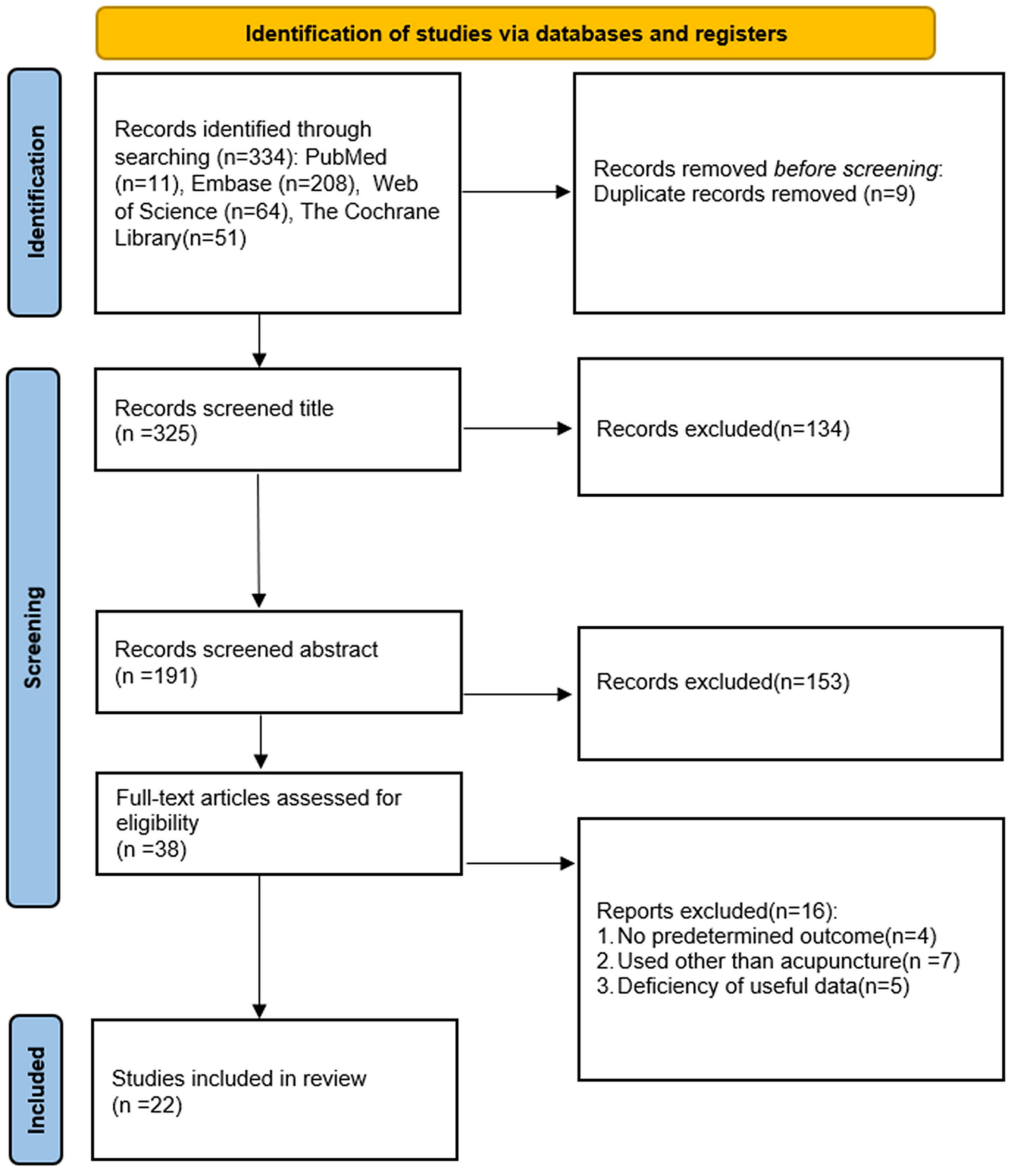
PRISMA statement flow chart.

Two independent researchers (YJH, WPC) retrieved a total of 334 studies in four databases. Firstly, 9 duplicates were removed, after reading the titles and abstracts, 287 studies that were not eligible for inclusion were excluded. Finally the full text was read to exclude 16 studies for the following reasons. A total of 22 studies (Lee et al., 2009; Kwon et al., 2012; Park et al., 2012; Yang et al., 2013; Guo et al., 2014; Le et al., 2016; Tanahashi et al., 2016; Zhang et al., 2016, 2020; Duan et al., 2016a,b; Jiang et al., 2018; Yue et al., 2018; Zhao et al., 2019; Luo et al., 2020; Mao et al., 2020; Xu et al., 2020; Jung et al., 2021; Li et al., 2021b; Chen et al., 2022; Wang et al., 2022) were finally included for meta-analysis (Figure 1).

### 3.2 Basic characteristics of the included study

A total of 22 studies with 497 animals were included, including 248 in the test group and 249 in the control group (Supplementary Table S1).

**Figure 2:**
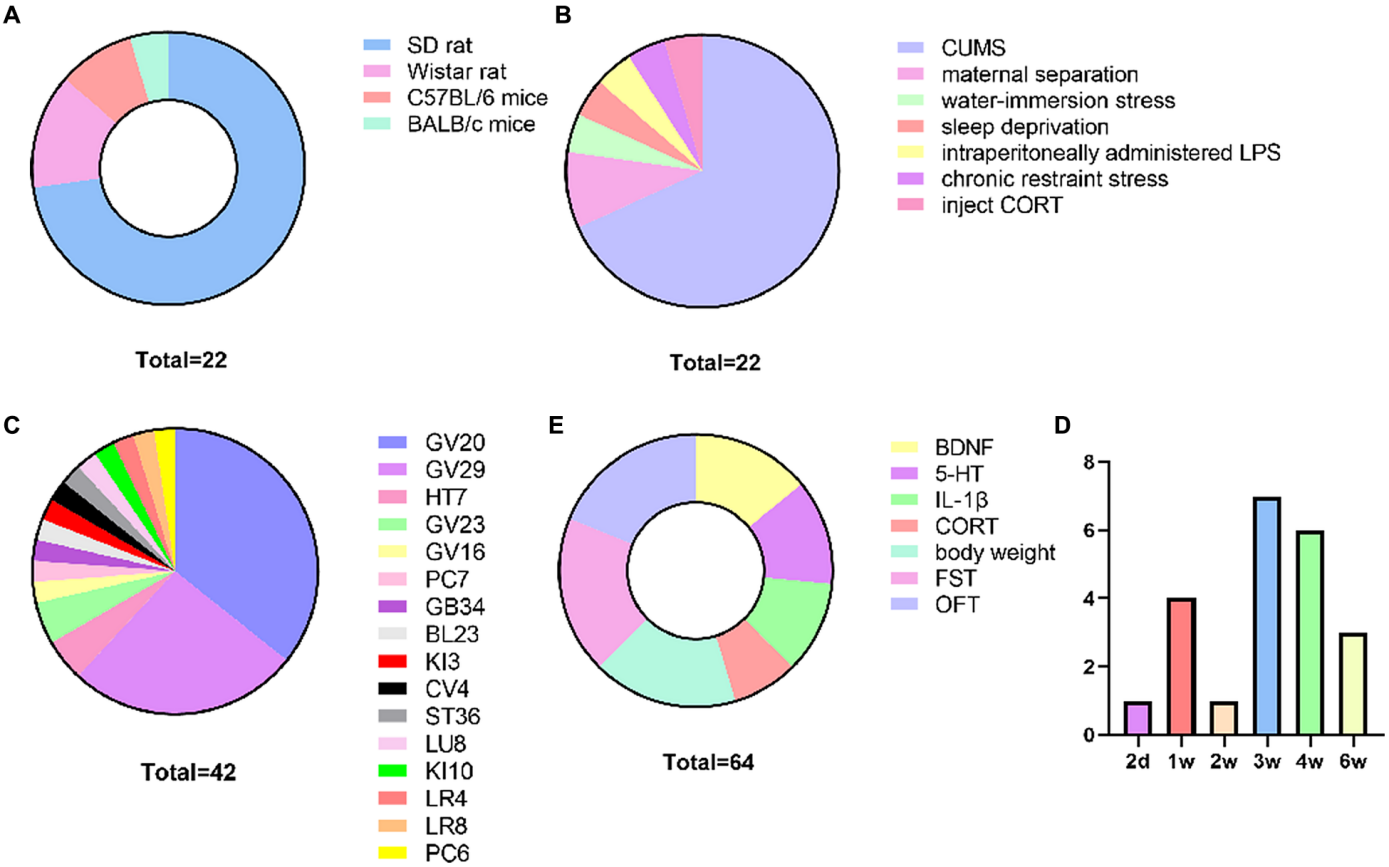
Basic characteristics of the included study. **(A)** Animal species, **(B)** Molding methods, **(C)** Frequency of acupoints use, **(D)** Courses of treatment, **(E)** Outcomes.

Animal species and modeling methods: all included studies reported the animal species and modeling methods. A total of 16 studies (Lee et al., 2009; Kwon et al., 2012; Park et al., 2012; Yang et al., 2013; Guo et al., 2014; Le et al., 2016; Zhang et al., 2016; Duan et al., 2016b; Jiang et al., 2018; Yue et al., 2018; Zhao et al., 2019; Luo et al., 2020; Mao et al., 2020; Li et al., 2021a; Chen et al., 2022) used SD rats, 3 studies (Tanahashi et al., 2016; Duan et al., 2016a; Zhang et al., 2020) used Wistar rats, 2 studies (Jung et al., 2021; Wang et al., 2022) used C57BL/6 mice, and 1 study (Xu et al., 2020) used BALB/c mice (Figure 2A). Modeling: 15 studies (Yang et al., 2013; Le et al., 2016; Zhang et al., 2016; Duan et al., 2016a; Jiang et al., 2018; Yue et al., 2018; Zhao et al., 2019; Luo et al., 2020; Mao et al., 2020; Li et al., 2021a,b; Chen et al., 2022; Wang et al., 2022, !!! INVALID CITATION!!!) used CUMS, 2 studies (Kwon et al., 2012; Park et al., 2012) used maternal separation, 1 study (Tanahashi et al., 2016) used water-immersion stress, 1 study (Xu et al., 2020) used sleep deprivation, 1 study (Zhang et al., 2020) used intraperitoneally administered LPS, 1 study (Jung et al., 2021) used chronic restraint stress, and 1 study (Lee et al., 2009) injected CORT Figure 2B.

Selection and combination of acupoints: the included studies used the following acupoints: GV20 (15), GV29 (11), GV23 (2), HT7 (2), and the frequency of the rest of the acupoints was 1 time. The most frequently used acupuncture point combination was GV20 + GV29. Figure 2C.

Courses of treatment: the courses of treatment was reported in the included studies and ranged from 2 days-6 weeks. The treatment courses in the included studies were concentrated in 3w (7), 4w (6), 1w (4), and 6w (3). Figure 2D.

Outcomes: BDNF, 5-HT, IL-1β, CORT, body weight, forced swimming test (FST), open field test (OFT): crossing number, rearing number. 9 studies (Park et al., 2012; Yang et al., 2013; Zhang et al., 2016; Duan et al., 2016b; Jiang et al., 2018; Luo et al., 2020; Mao et al., 2020; Xu et al., 2020; Li et al., 2021a) mentioned BDNF, 8 studies (Kwon et al., 2012; Park et al., 2012; Le et al., 2016; Duan et al., 2016a,b; Zhao et al., 2019; Zhang et al., 2020; Li et al., 2021a) mentioned 5-HT, 7 studies (Guo et al., 2014; Yue et al., 2018; Zhang et al., 2020; Jung et al., 2021; Li et al., 2021b; Chen et al., 2022; Wang et al., 2022) mentioned IL-1β, 5 studies (Lee et al., 2009; Kwon et al., 2012; Le et al., 2016; Tanahashi et al., 2016; Jung et al., 2021) mentioned CORT, 11 studies (Yang et al., 2013; Guo et al., 2014; Tanahashi et al., 2016; Duan et al., 2016a,b; Jiang et al., 2018; Zhao et al., 2019; Luo et al., 2020; Jung et al., 2021; Li et al., 2021a; Chen et al., 2022) mentioned body weight, 12 studies (Lee et al., 2009; Yang et al., 2013; Le et al., 2016; Tanahashi et al., 2016; Duan et al., 2016a,b; Yue et al., 2018; Xu et al., 2020; Zhang et al., 2020; Jung et al., 2021; Chen et al., 2022; Wang et al., 2022) mentioned FST, 12 studies (Guo et al., 2014; Le et al., 2016; Zhang et al., 2016, 2020; Duan et al., 2016a; Yue et al., 2018; Zhao et al., 2019; Luo et al., 2020; Jung et al., 2021; Li et al., 2021b; Chen et al., 2022) mentioned OFT (Figure 2E).

### 3.3 Risk of bias

**Figure 3:**
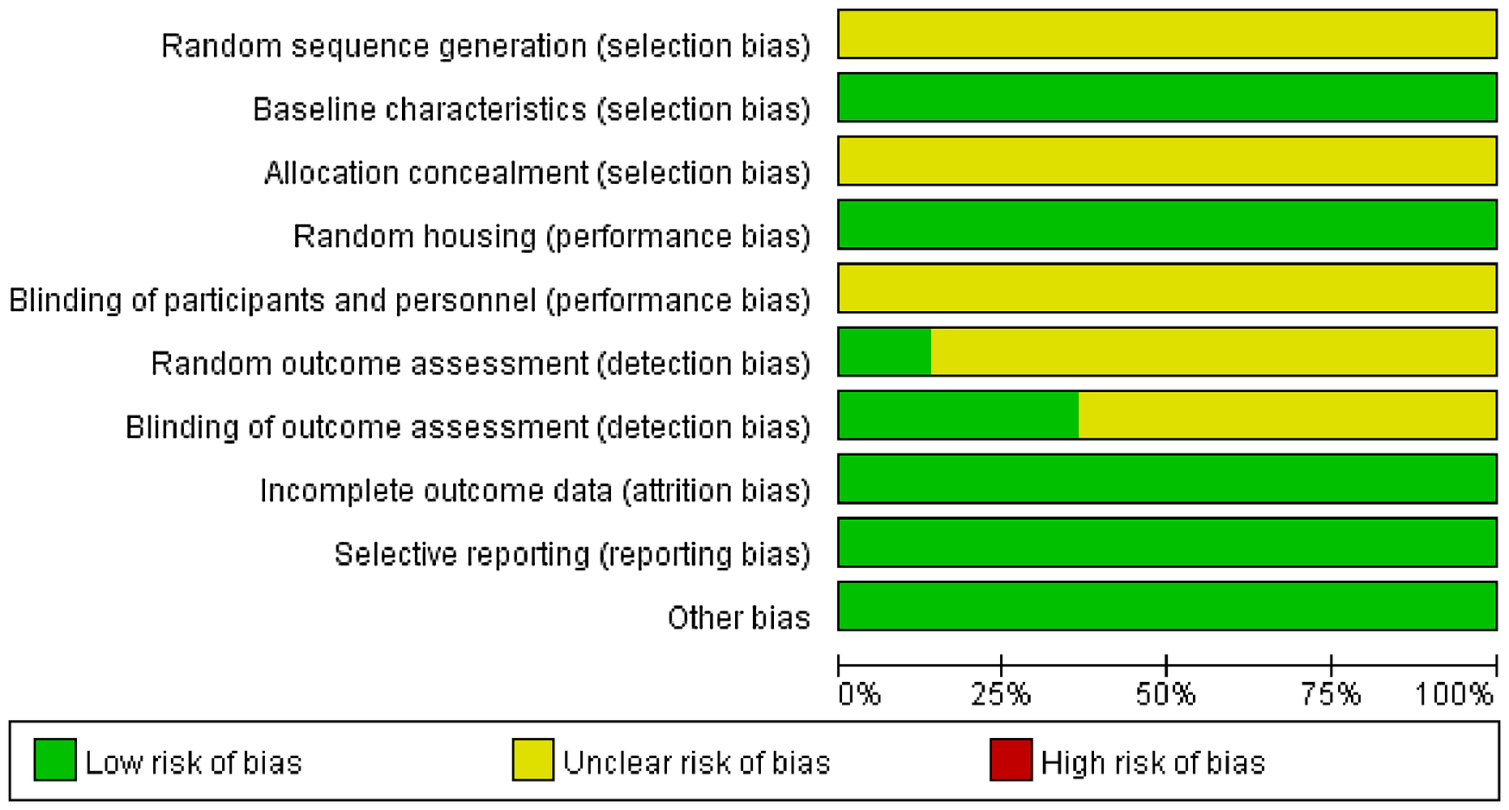
Risk of bias.

The risk of bias for the studies according to the SYRCLE risk assessment tool showed that all studies included were randomly assigned, but the method of random assignment was not specifically described. In all studies, the basic characteristics of the animals were not significantly different. All studies did not describe the method of random housing, but all specifically described the house environment, with no significant differences. Blinding of the experimenter was not possible because the test groups were acupuncture. 3 studies (Yang et al., 2013; Jung et al., 2021; Wang et al., 2022) used random outcome assessment. 8 studies (Guo et al., 2014; Le et al., 2016; Zhang et al., 2016; Jiang et al., 2018; Yue et al., 2018; Li et al., 2021a,b) used blinding of outcome assessment. All studies had complete outcome data with no selective reporting and other bias (Figure 3 and Supplementary Figure S1).

### 3.4 Meta-analysis

#### 3.4.1 Body weight

**Figure 4:**
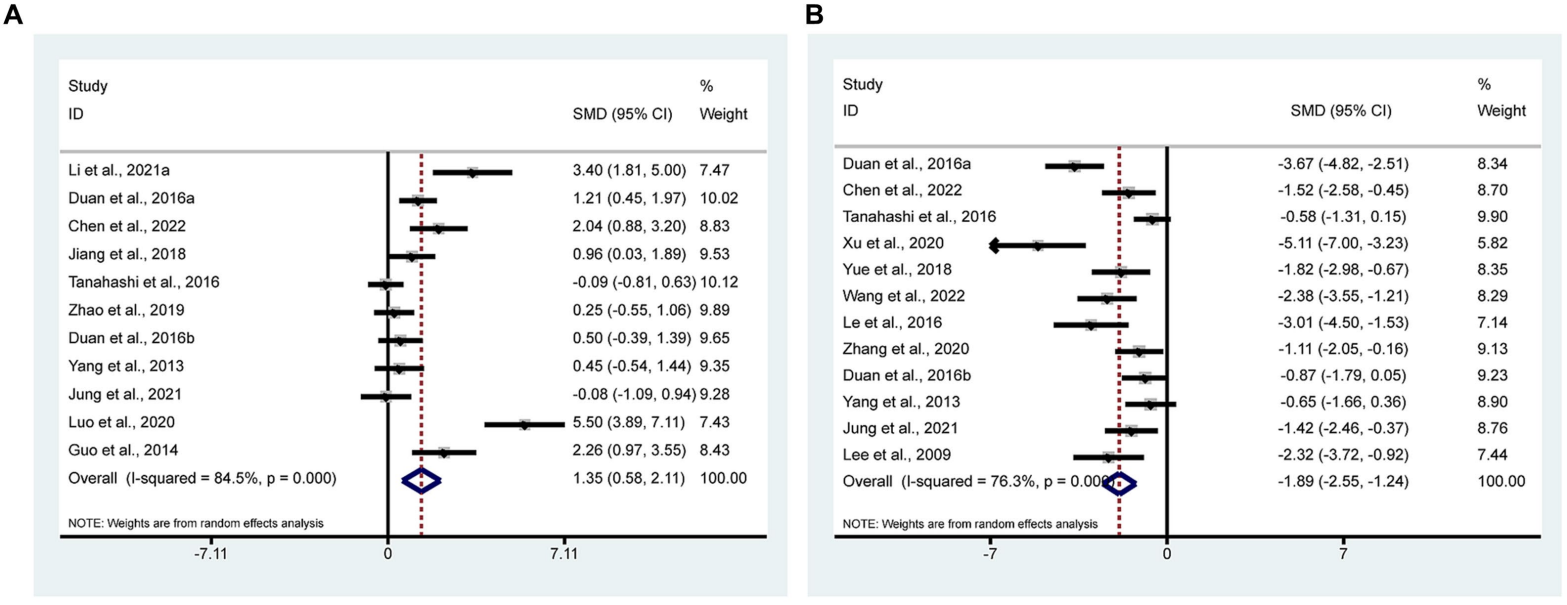
Forest plots of meta-analysis. **(A)** Body weight; **(B)** FST.

Appetite in depression usually decreases and so does body weight, which may reflect the efficacy of the treatment. 11 studies (Yang et al., 2013; Guo et al., 2014; Tanahashi et al., 2016; Duan et al., 2016a,b; Jiang et al., 2018; Zhao et al., 2019; Luo et al., 2020; Jung et al., 2021; Li et al., 2021a; Chen et al., 2022) with 237 animals involved in body weight. According to the meta-analysis, the test group had significantly greater body weight than the control group [SMD = 1.35, 95% CI (0.58, 2.11); *I*^2^ = 84.5%] (*p* < 0.05). It indicates that acupuncture can significantly increase the body weight of animal models of depressive-like behaviors (Figure 4A).

#### 3.4.2 FST

FST is one of the behavioral test of animal models of depression, which can reflect the efficacy of interventions. 12 studies (Lee et al., 2009; Yang et al., 2013; Le et al., 2016; Tanahashi et al., 2016; Duan et al., 2016a,b; Yue et al., 2018; Xu et al., 2020; Zhang et al., 2020; Jung et al., 2021; Chen et al., 2022; Wang et al., 2022) with 241 animals involved in FST. According to the meta-analysis, the test group’s FST was significantly higher than that of the control group’s [SMD = −1.89, 95% CI (−2.55, −1.24); *I*^2^ = 76.3%] (*p* < 0.05). It indicates that acupuncture can significantly improve FST in animal models of depressive-like behaviors (Figure 4B).

#### 3.4.3 OFT

**Figure 5:**
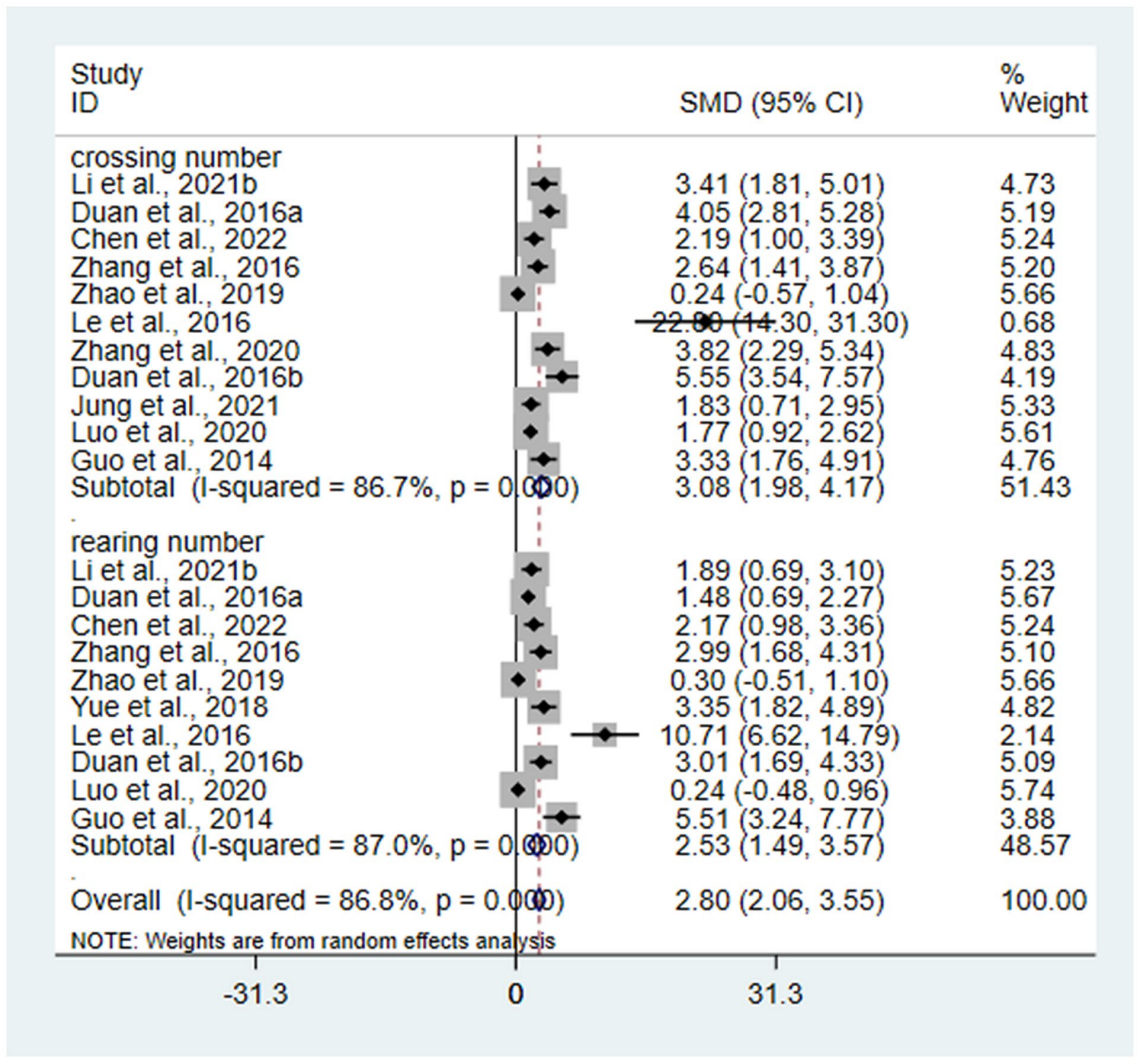
Forest plots of meta-analysis of OFT.

OFT is one of the main behavioral test of animal models of depression and may reflect the efficacy of interventions. 12 studies (Guo et al., 2014; Le et al., 2016; Zhang et al., 2016, 2020; Duan et al., 2016a,b; Yue et al., 2018; Zhao et al., 2019; Luo et al., 2020; Jung et al., 2021; Li et al., 2021b; Chen et al., 2022) with 246 animals involved in OFT. According to the meta-analysis, the test group had better crossing and rearing numbers than the control group [SMD = 3.08, 95% CI (1.98, 4.17); *I*^2^ = 86.7%], [SMD = 2.53, 95% CI (1.49, 3.57); *I*^2^ = 87.0%] (*p* < 0.05). It indicates that acupuncture significantly improves OFT in animal models of depressive-like behaviors (Figure 5).

#### 3.4.4 BDNF

**Figure 6:**
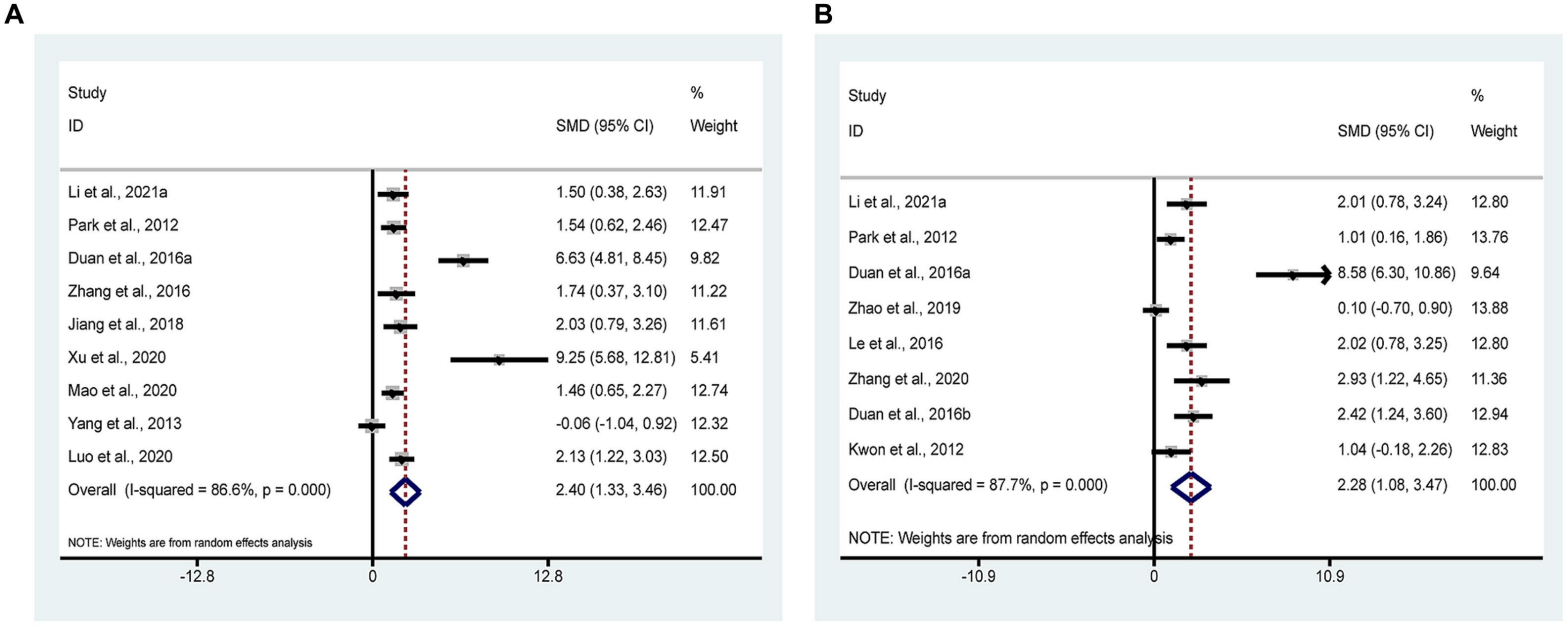
Forest plots of meta-analysis. **(A)** BDNF; **(B)** 5-HT.

BDNF is an important member of antidepressant neurotrophic factors with growth and developmental effects on neurons. 9 studies (Park et al., 2012; Yang et al., 2013; Zhang et al., 2016; Duan et al., 2016b; Jiang et al., 2018; Luo et al., 2020; Mao et al., 2020; Xu et al., 2020; Li et al., 2021a) with 192 animals involved in BDNF. According to the meta-analysis, the test group had statistically increased BDNF compared to the control group [SMD = 2.40, 95% CI (1.33, 3.46); *I*^2^ = 86.6%] (*p* < 0.05). It indicates that acupuncture can significantly increase BDNF in animal models of depressive-like behaviors (Figure 6A).

#### 3.4.5 5-HT

5-HT is a monoamine neurotransmitter, responsible for neural message transmission, and is an important object of neurotransmitter mechanisms in depression. 8 studies (Kwon et al., 2012; Park et al., 2012; Le et al., 2016; Duan et al., 2016a,b; Zhao et al., 2019; Zhang et al., 2020; Li et al., 2021a) with 156 animals involved in 5-HT. The meta-analysis revealed that the test group had significantly more 5-HT than the control group [SMD = 2.28, 95% CI (1.08, 3.47); *I*^2^ = 87.7%] (*p* < 0.05). It indicates that acupuncture can significantly increase 5-HT in animal models of depressive-like behaviors (Figure 6B).

#### 3.4.6 Il-1β

**Figure 7:**
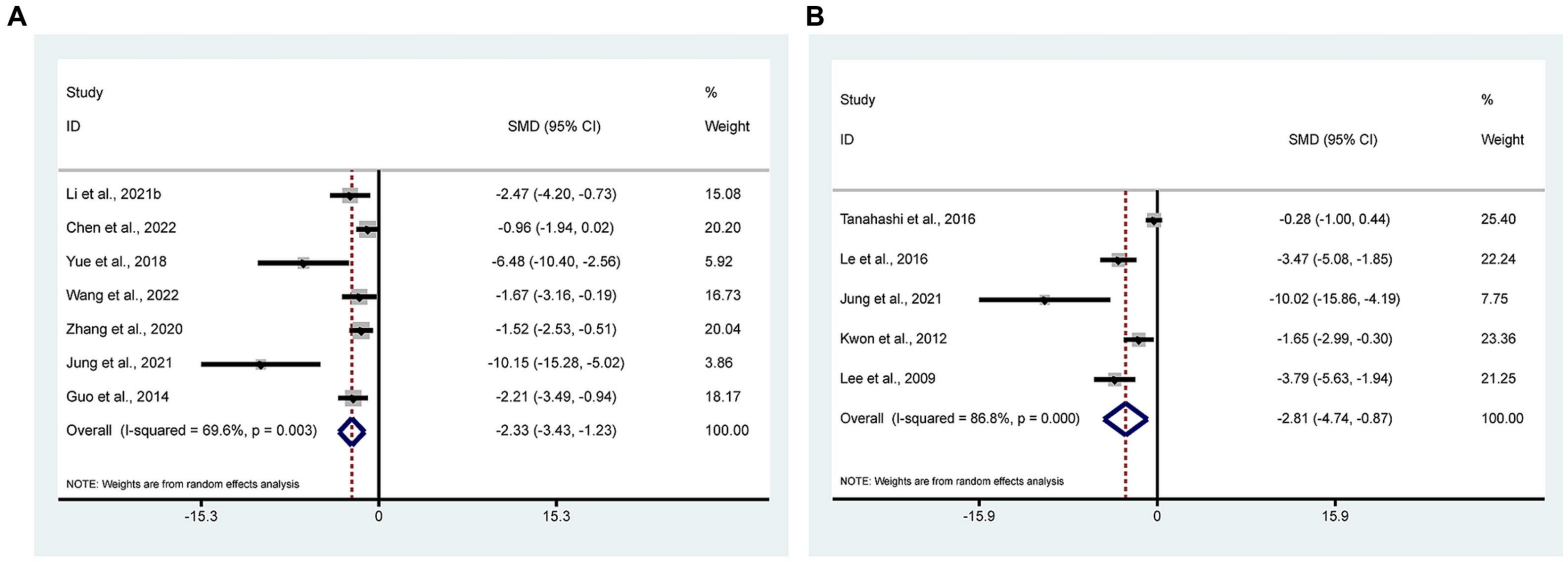
Forest plots of meta-analysis. **(A)** IL-1β; **(B)** CORT.

IL-1β is an indicator of inflammation, a pro-inflammatory cytokine belonging to the immunoinflammatory mechanism of depression, which can cause central inflammation and promote the onset and progression of depression. 7 studies (Guo et al., 2014; Yue et al., 2018; Zhang et al., 2020; Jung et al., 2021; Li et al., 2021b; Chen et al., 2022; Wang et al., 2022) with 92 animals involved in IL-1β. According to the meta-analysis, the test group had lower IL-1β levels compared to the control group [SMD = −2.33, 95% CI (− 3.43, −1.23); I2 = 69.6%], which was statistically significant (*p* < 0.05). It indicates that acupuncture can significantly reduce IL-1β in animals with depressive-like behaviors (Figure 7A).

#### 3.4.7 CORT

Depression is triggered by a significant increase in HPA axis activity, which leads to elevated CORT levels. 5 studies (Lee et al., 2009; Kwon et al., 2012; Le et al., 2016; Tanahashi et al., 2016; Jung et al., 2021) with 80 animals involved in CORT. According to the meta-analysis, the test group had significantly lower CORT than the control group [SMD = −2.81, 95% CI (−4.74, −0.87); *I*^2^ = 86.8%] (*p* < 0.05). It indicates that acupuncture can significantly reduce CORT in animal models of depressive-like behaviors (Figure 7B).

### 3.5 Subgroup meta-analysis

#### 3.5.1 Body weight

Subgroup meta-analysis of the different acupuncture methods (MA, EA) in the test group showed that the body weight of MA, EA was heavier than that of the control group [SMD = 1.12, 95% CI (0.01, 2.23); *I*^2^ = 82.9%], [SMD = 1.55, 95% CI (0.42, 2.69); *I*^2^ = 87.4%], with statistically significant differences (*p* < 0.05). The result of the different courses showed that the body weight of the test group was heavier than that of the control group at 4w and 6w (*p* < 0.05). Meanwhile, the result of the modeling methods and species of animals showed that the body weight of CUMS was significantly heavier than that of the control group in the grouping of modeling method (*p* < 0.05), and in the grouping of species, compared to the control group, the body weight of Wistar rats was not statistically significant (*p* > 0.05). This may be due to the small sample size. The results of subgroup analysis were consistent with the meta results, which indicated that the results of the study were not significantly affected by the methods and duration of acupuncture (Supplementary Table S2).

#### 3.5.2 FST

The different acupuncture methods (MA, EA) in the test group were analyzed in a subgroup meta-analysis. The result showed that FST was significantly better in MA, EA than in the control group [SMD = −1.31, 95% CI (−2.02, −0.61); *I*^2^ = 47.4%], [SMD = −2.19, 95% CI (−3.13, −1.26); *I*^2^ = 80.7%] (*p* < 0.05). The result of the different sessions showed that the test group had better FST than the control group at 1w, 2w, 3w, and 4w (*p* < 0.05). Meanwhile, subgroup meta-analysis was performed on the modeling methods and species of animals, and the results showed that the FST of the test group was significantly better than that of the control group (*p* < 0.05). The results of subgroup analysis were consistent with the meta results, which indicated that the results of the study were not significantly affected by the methods of acupuncture, treatment, modeling methods, and species (Supplementary Table S2).

#### 3.5.3 BDNF

The different acupuncture methods (MA, EA) in the test group were analyzed in a subgroup meta-analysis. Subgroup Meta-analysis showed that BDNF was significantly higher in MA, EA than in the control group [SMD = 1.66, 95% CI (1.10, 2.23); *I*^2^ = 0.0%], [SMD = 3.36, 95% CI (1.29, 5.43); *I*^2^ = 93.2%] (*p* < 0.05). A subgroup meta-analysis of the different courses showed that the BDNF was higher in the test group than in the control group at 1-3w and 4-6w (*p* < 0.05). Additionally, subgroup meta-analysis were conducted on the modeling methods and species. The results showed that the test group’s BDNF was significantly higher than the control group’s BDNF (*p* < 0.05). The results of subgroup meta-analysis were consistent with the meta results, which indicated that the results of the study were not significantly affected by the methods of acupuncture, courses, modeling methods, and species (Supplementary Table S2).

#### 3.5.4 5-HT

The different acupuncture methods (MA, EA) in the test group were analyzed in a subgroup meta-analysis. Subgroup meta-analysis showed that 5-HT was significantly higher in MA, EA than in the control group [SMD = 1.26, 95% CI (0.66, 1.87); *I*^2^ = 0.0%], [SMD = 3.03, 95% CI (0.90, 5.15); *I*^2^ = 92.6%] (*p* < 0.05). A subgroup meta-analysis of the different courses showed that 5-HT was higher in the test group than in the control group at 1-2w and 4-6w (*p* < 0.05). Meanwhile, subgroup meta-analysis of the modeling methods and species of the animals showed that the test group had significantly more 5-HT than the control group (*p* < 0.05). The results of subgroup analysis were consistent with the meta results, which indicated that the results of the study were not significantly affected by the methods of acupuncture, courses, modeling methods, and species (Supplementary Table S2).

#### 3.5.5 Il-1β

The different acupuncture methods (MA, EA) in the test group were analyzed in a subgroup meta-analysis. Subgroup meta-analysis showed that IL-1β was significantly lower in MA, EA than in the control group [SMD = −3.30, 95% CI (−6.27, −0.33); *I*^2^ = 84.9%], [SMD = −2.10, 95% CI (−3.19, −1.02); *I*^2^ = 51.0%] (*p* < 0.05). The subgroup meta-analysis of the courses of treatment in the test group showed that the test group’s IL-1β was lower than the control group’s IL-1β at 1-3w and 4-6w (*p* < 0.05). At the same time, subgroup meta-analysis of the modeling methods and species of animals showed that in the modeling methods, the test group had significantly lower IL-1β than the control group (*p* < 0.05). The IL1-β of C57BL/6mice was not statistically significant compared with the control group in the subgroup analysis of the species (*p* > 0.05). The reason may be that the sample size was small and only 2 studies (Jung et al., 2021; Wang et al., 2022) involved IL-1β. The results of subgroup meta-analysis were consistent with the meta results, which indicates that the results of the study were not significantly affected by the methods of acupuncture, courses, and modeling methods (Supplementary Table S2).

#### 3.5.6 CORT

The different acupuncture methods (MA, EA) in the test group were analyzed in a subgroup meta-analysis. The result showed that MA, EA had significantly lower CORT than the control group [SMD = −2.64, 95% CI (−4.86, −0.42); *I*^2^ = 86.8%], [SMD = −3.47, 95% CI (−5.08, −1.85)], the difference was statistically significant (*p* < 0.05). The result of the different courses showed that the test group’s CORT was lower than the control group’s CORT at 1–2 w (*p* < 0.05), but at 3 w, the test group and the control group was not statistically significant (*p* > 0.05), probably due to the small sample size. Meanwhile, subgroup meta-analysis of the modeling methods and species of animals showed that the CORT of the test group was significantly lower than that of the control group (*p* < 0.05). The results of subgroup analysis were consistent with the meta results, which indicated that the results of the study were not significantly affected by the methods of acupuncture, modeling methods, and species (Supplementary Table S2).

### 3.6 Sensitivity analysis

Sensitivity analysis of BDNF, 5-HT, IL-1β, CORT, body weight, FST, and OFT showed that the sensitivity analysis of all outcomes was stable and no significant bias was found, indicating that the meta-analysis results were stable (*p* < 0.05) (Supplementary Figure S2).

### 3.7 Publication bias

The inverted funnel plot and Egger analysis were used to detect publication bias. Egger analysis showed that the publication bias for BDNF, 5-HT, IL-1β, CORT, body weight, FST, and OFT were not statistically significant (*p* < 0.05), indicating that there may be publication bias in these studies (Supplementary Figure S3).

### 3.8 Certainty assessment

The GRADE protocol was used to assess the certainty of the evidence (Supplementary Table S3). Accordingly, the evidence for BDNF, 5-HT, IL-1β, CORT, body weight, FST, and OFT were graded as low due to inconsistencies or imprecision.

## 4 Discussion

A total of 22 studies and 497 animals were included in the meta-analysis. Meta-analysis showed that acupuncture increased BDNF, 5-HT, decreased CORT, IL-1β, and improved behavioral tests of depressive-like behaviors (FST, OFT) by increasing body weight (*p* < 0.05) to treat animals with depressive-like behaviors compared with the control group.

To our knowledge, a meta-analysis examined the effectiveness of acupuncture on depressive-like behaviors in animals, where acupuncture improved OFT, sucrose intake test, final weight and gain weight in animal models of depressive-like behaviors (Kou et al., 2017). It is consistent with this meta-analysis regarding behavioral tests, body weight, indicating the efficacy of acupuncture on animal models of depressive-like behaviors. However, there is no systematic evaluation of the indicators related to the mechanism of acupuncture for depression in animal models. Consequently, we analyzed the effects of acupuncture on animals with depressive-like behaviors.

### 4.1 Neurotrophins

**Figure 8:**
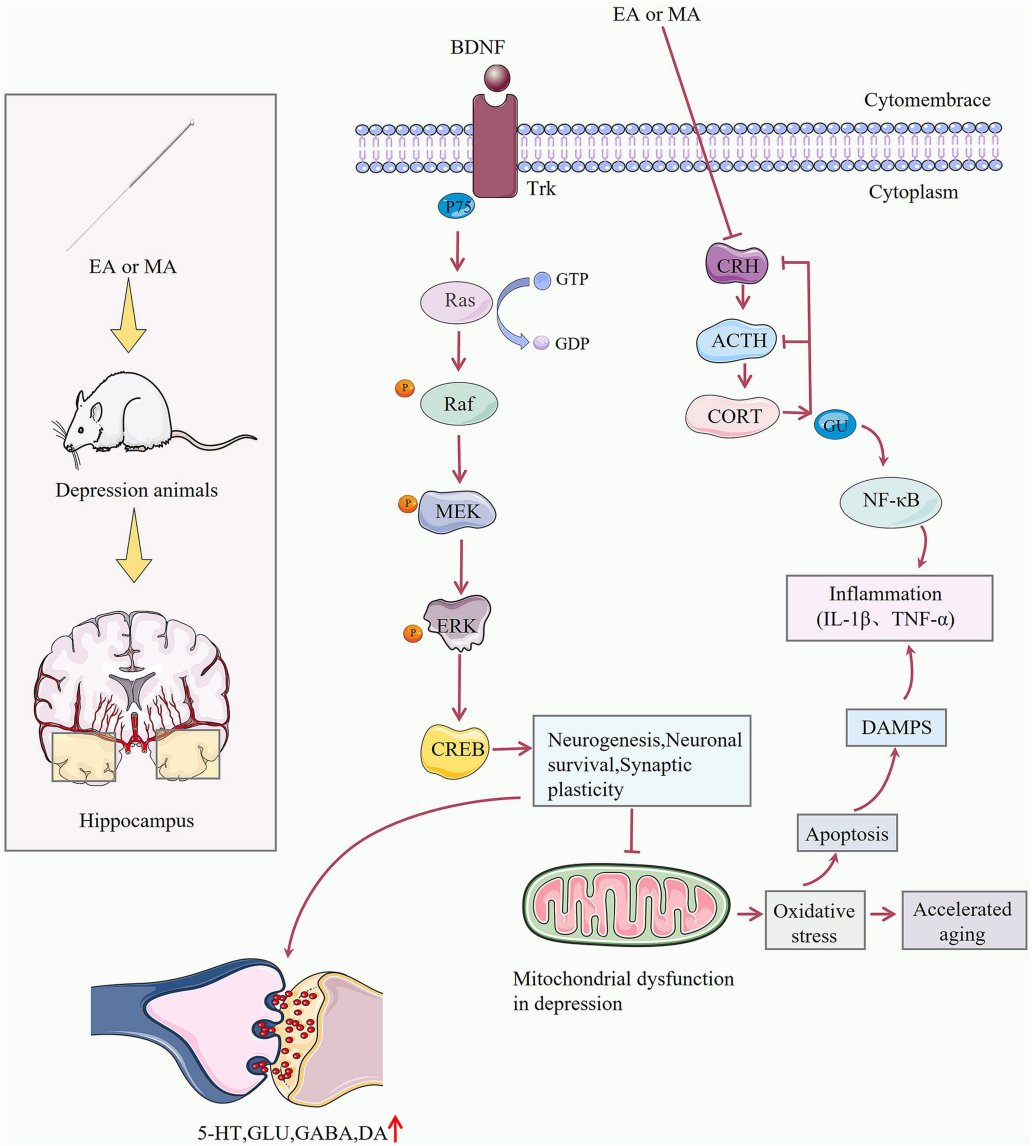
The main mechanism of acupuncture in animal models of depressive-like behaviors. Trk, telomerin-related kinase; P75, P75 neurotrophic factor receptor; GDP, guanosine diphosphate; GTP, guanosine Triphosphate; MEK, Mitogen-activated protein kinase; ERK, Extracellular signal-regulated kinase; CREB, cAMP-response element binding protein; CRH, corticotropin releasing hormone; ACTH, adrenocorticotrophic hormone; GR, glucocorticoid receptor; NF-kB, nuclear factor-kB; DAMPS, damage associated molecular patterns; GLU, glutamic acid; GABA, γ-aminobutyric acid; DA, Dopamine.

Neurotrophins have important roles in neuronal growth, development, and survival. BDNF as an important member of neurotrophins, can activate p-telomerin-related kinase (Trk) and p75 receptors, which are closely related to the Ras-MAPK–ERK pathway (Fries et al., 2023). Evidence suggests that depressed patients have lower neurotrophins levels, especially in persistently depressed and severely depressed patients with decreased blood BDNF levels (Bus et al., 2015; Kishi et al., 2017), The animal models of depressive-like behaviors also have decreased BDNF (Tayyab et al., 2018), suggesting that a decrease in BDNF may contribute to depressive-like behaviors. Clinical studies have shown (Sun et al., 2013) that electroacupuncture has a faster onset of action and response rate than fluoxetine to produce neurotrophins to improve depression. By stimulating the Ras-MAPK–ERK pathway and increasing BDNF protein expression, acupuncture may alleviate depression (Sun et al., 2014). Meta-analysis showed that acupuncture increased BDNF compared to the control groups. Acupuncture may have an antidepressant effect by increased BDNF levels in animal models of depressive-like behaviors (Figure 8).

### 4.2 Neurotransmitters

Neurotransmitters are specific chemicals released from nerve endings and are responsible for the transmission of neural messages. Currently, monoamine neurotransmitters (5-HT, DA, NE) and amino acid neurotransmitters (GABA, GLU) are widely studied. Among the studies of monoamine neurotransmitters, the study of 5-HT is particularly important (Bannerman et al., 2014; Mouri et al., 2016). It had been demonstrated that depression was associated with reduced 5-HT release and decreased synaptic gap 5-HT levels following the diminished function of the 5-HT system (Morrissette and Stahl, 2014). A clinical study found (Yang et al., 2020) that acupuncture can alleviate symptoms of despair and feelings of anxiety in depression. Meanwhile, animal experiments found that acupuncture increased serum 5-HT levels in rats with depressive-like behaviors and alleviated the decrease in 5-HT and 5-HT transporter protein expression levels in the hippocampus of depressive-like behaviors in rats (Shu et al., 2013). Meta-analysis showed that acupuncture significantly increased 5-HT levels compared to the control groups, so it was speculated that the effect of acupuncture on 5-HT neurotransmitters may be an important mechanism for its antidepressant effect.

### 4.3 Immunoinflammation

According to recent research, depressed patients’ immune systems may be abnormally activated because inflammatory cytokines are secreted abnormally (Liu et al., 2020). The inflammatory cytokine are significantly different before and after antidepressant treatment. Indeed, pro-inflammatory cytokines (IL-1β) are strongly associated with the development of depression (Petralia et al., 2020; Orsolini et al., 2022). Depression may be caused by an increase in permeability of the blood–brain barrier, triggering inflammation in the brain (Ohkusa and Koido, 2015). Meta-analysis showed that acupuncture significantly reduced IL-1β levels in animal models of depressive-like behaviors, and the mechanism may be that acupuncture reduces pro-inflammatory cytokines and alleviates central inflammation, which may have a therapeutic effect on animals with depressive-like behaviors.

### 4.4 Neuroendocrine

Some studies (Baudry et al., 2013) had shown a significant association between neuroendocrine alterations and certain features of depression, and most scholars believe that the hypothalamic–pituitary–adrenal (HPA) axis is widely believed to play a role in the development of depression (Yano et al., 2015). Studies had shown that depression was triggered by significantly increased HPA axis activity, elevated serum adrenocorticotropin-releasing hormone (ACTH) and CORT levels in patients with depression (Lupien et al., 2009). Some studies showed (Yang et al., 2020) that acupuncture reduced CORT levels, inhibits the HPA axis, and improves depressive-like behaviors. In addition, depression occurs in relation to the function of the hypothalamic–pituitary-thyroid axis (HPT axis) and hypothalamic–pituitary-gonadal axis (HPG axis).

In the treatment of depressive-like behaviors in animals with acupuncture, we had also focused on the influence of acupoint and acupuncture site on the efficacy and mechanism. By statistics, GV20 and GV29 were used more frequently, with the most frequent use of head acupoints, concentrated in the Governor Vessel (GV), followed by hand acupoints. Electroacupuncture GV20 and GV29 improved behavioral tests of depressive-like behaviors (Guo et al., 2014; Mo et al., 2014), while manual acupuncture GV20 and GV29 also improved behavioral tests, suggested that acupuncture GV20 and GV29 have unique antidepressant effects, regardless of the acupuncture method (Lu et al., 2013; Jung et al., 2015). In addition, HT7 (hand acupoint) has been used for depression in several clinical studies (Röschke et al., 2000; Han et al., 2002), and acupuncture of HT7 modulated the HPA axis (Park et al., 2011, 2012). In regulating neurotransmitters and intestinal biological mechanisms, GV23 (head acupoint) and PC7 (hand acupoint) played an important role in increasing 5-HT (Huang et al., 2019; Li et al., 2021a). GV16 and GV23 also belong to head acupoints, and in previous studies (Cheng et al., 2021; Chen et al., 2022), they reduced inflammatory levels such as IL-1β and improved the behavior test of animal models of depressive-like behaviors. Although most of the studies confirmed the effectiveness of acupuncture in treating depression, we also paid attention to some negative studies and explored the reasons. Some clinical studies indicated that there was no significant difference in improving the scores of depressed patients in the comparison of electroacupuncture GV20, GV29 and electroacupuncture non-acupuncture points (Allen et al., 2006; Andreescu et al., 2011; Kim et al., 2022). Meanwhile, in an animal experiment, acupuncture GV20 or GV29 did not alleviate depression in rats (Takagi et al., 2017) These negative results may be due to the control of the placebo effect of sham acupuncture in the control group, the frequency dose of the intervention, and the fact that the use of only GV20, GV29 may not achieve the desired efficacy. For example, in some clinical studies (Luo et al., 1985), acupuncturists needled GV20, GV29 and improved symptoms in patients with depression, while another clinical trial (Andreescu et al., 2011) similarly needled GV20, GV29, but the results were negative, probably due to the difference in intervention dose. In clinical practice, acupuncturists increase the selection of acupoints and frequency of interventions according to the constitution, individualized symptoms, and pulse of depressed patients, and treatment protocols for individual acupoints are less common. Thus, the efficacy of acupuncture in the treatment of depression is not through the action of one or two acupoints, but rather the synergistic action of the appropriate acupoints, the frequency of interventions, and the dosage, among other factors. In conclusion, head acupoints were preferred for antidepressant clinical and experimental use; acupuncture and acupoints played an important role in multiple mechanisms of antidepressant disorders and facilitate the improvement of depression.

We tried to find sources of heterogeneity by subgroup meta-analysis, sensitivity analysis, but could not find heterogeneity. In the study of animal models, we excluded the effects of animal species, modeling method, acupuncture methods, and courses. Also, we focused on the important influence of acupoints and electroacupuncture parameters in acupuncture for depression, thus possibly leading to heterogeneity. GV20 and GV29 are the more commonly used acupoints in studies of acupuncture interventions for animals with depressive-like behaviors, and these two acupoints were selected for the most frequent use in the studies we included. However, due to the large variation in the acupoints used among studies, it was not possible to look for heterogeneity by subgroup analysis, this could be one of the sources of heterogeneity. It is also worth noting that the parameters of electroacupuncture (waveform, frequency, voltage) are also important factors that influence the efficacy. 1 study concluded that in the treatment of animals with depressive-like behaviors, the parameters of electroacupuncture were appropriate for stimulation with low frequency, sparse waves, retention time of about 30 min, and low to medium intensity (2 Hz) (Yanhong et al., 2011). Another study concluded that when a single electroacupuncture treatment was chosen, the frequency of 100 Hz was more effective in relieving depressive symptoms than 2 Hz, while a stimulation frequency of 2 Hz combined with low-dose antidepressants was more effective in relieving symptoms than a single treatment (Park et al., 2011; Wang et al., 2015). In the studies we included, these electroacupuncture parameters were heterogeneous and there were no relevant uniform regulations. Therefore, more studies are needed to standardize the determination of acupoints selection, site selection, and electroacupuncture parameters for depression.

### 4.5 Limitations

(1) The meta-analysis had high heterogeneity, although our attempts to find the source of heterogeneity failed, which may affect the accuracy of the results; (2) The method of random assignment was not specifically described in the included studies, which may lead to the risk of bias; (3) There were not enough studies on the indicators related to the mechanism of animals with depressive-like behaviors treated by acupuncture to fully describe the effect of acupuncture on the mechanism of depression; (4) Due to the limitation of language, related studies could not be fully included; (5) The current studies on depression mechanisms are independent and lack the connection between mechanisms, which cannot reflect the relationship between different mechanisms of acupuncture anti-depression.

### 4.6 Conclusion

Acupuncture may treat animal models of depressive-like behaviors through a variety of mechanisms including regulation of neurotrophins, neurotransmitters, inflammatory cytokines, and neuroendocrine system. However, more high-quality, large-sample, multi-mechanism studies are needed to verify this conclusion in the future.

## Data availability statement

The original contributions presented in the study are included in the article/Supplementary material, further inquiries can be directed to the corresponding authors.

## Author contributions

YH: Conceptualization, Data curation, Formal analysis, Investigation, Methodology, Software, Writing – original draft. WC: Conceptualization, Data curation, Formal analysis, Investigation, Methodology, Writing – original draft. XL: Conceptualization, Data curation, Formal analysis, Methodology, Supervision, Writing – original draft. TT: Conceptualization, Investigation, Writing – original draft. TW: Data curation, Methodology, Writing – original draft. SQ: Conceptualization, Data curation, Formal analysis, Writing – original draft. GL: Data curation, Supervision, Writing – original draft. CY: Funding acquisition, Resources, Supervision, Validation, Visualization, Writing – review & editing. ML: Supervision, Validation, Visualization, Writing – review & editing. LD: Conceptualization, Funding acquisition, Methodology, Project administration, Resources, Supervision, Validation, Visualization, Writing – review & editing.

## Footnotes

^1^ http://www.prisma-statement.org

^2^ gradeworkinggroup.org
