# Supplementary material for: Efficacy and mechanism of acupuncture in animal models of depressive-like behaviors: a systematic review and meta-analysis
Source: Front Neurosci. 2024 Feb 15;18:1330594. doi: 10.3389/fnins.2024.1330594 (PMC10902032; doi:10.3389/fnins.2024.1330594)
Supplement: Supplementary file 1 [file Data_Sheet_1.docx]

Supplementary Material

**1 Appendix 1.** Search strategies.

**2 Supplementary Figure 1.** Risk of bias assessment.

**3 Supplementary Figure 2.** The sensitivity analysis.

**4 Supplementary Figure 3.** The publication bias.

# **Appendix 1.** Search strategies.

**Pubmed**

(((((("Rabbits"[Mesh]) OR (Rabbit[Title/Abstract] OR New Zealand Rabbits[Title/Abstract] OR Rabbit, New Zealand[Title/Abstract] OR Rabbits, New Zealand[Title/Abstract] OR Zealand Rabbit, New[Title/Abstract] OR Zealand Rabbits, New[Title/Abstract] OR New Zealand White Rabbit[Title/Abstract] OR New Zealand Rabbit[Title/Abstract] OR New Zealand White Rabbits[Title/Abstract] OR NZW Rabbits[Title/Abstract] OR NZW Rabbit[Title/Abstract] OR Rabbit, NZW[Title/Abstract] OR Rabbits, NZW[Title/Abstract] OR Rabbit, Domestic[Title/Abstract] OR Domestic Rabbit[Title/Abstract] OR Domestic Rabbits[Title/Abstract] OR Rabbits, Domestic[Title/Abstract] OR Oryctolagus cuniculus[Title/Abstract] OR cuniculus, Oryctolagus[Title/Abstract] OR Belgian Hare[Title/Abstract] OR Hare, Belgian[Title/Abstract] OR Chinchilla Rabbits[Title/Abstract] OR Chinchilla Rabbit[Title/Abstract] OR Rabbit, Chinchilla[Title/Abstract] OR Rabbits, Chinchilla[Title/Abstract])) OR (("Mice"[Mesh]) OR (Mus[Title/Abstract] OR Mouse[Title/Abstract] OR Mus domesticus[Title/Abstract] OR Mus musculus domesticus[Title/Abstract] OR domesticus, Mus musculus[Title/Abstract] OR Mus musculus[Title/Abstract] OR Mice, House[Title/Abstract] OR House Mice[Title/Abstract] OR Mouse, House[Title/Abstract] OR House Mouse[Title/Abstract] OR Mouse, Swiss[Title/Abstract] OR Swiss Mouse[Title/Abstract] OR Swiss Mice[Title/Abstract] OR Mice, Swiss[Title/Abstract] OR Mice, Laboratory[Title/Abstract] OR Laboratory Mice[Title/Abstract] OR Mouse, Laboratory[Title/Abstract] OR Laboratory Mouse[Title/Abstract]))) OR (("Rats"[Mesh]) OR (Rat[Title/Abstract] OR Rattus[Title/Abstract] OR Rattus norvegicus[Title/Abstract] OR Rats, Norway[Title/Abstract] OR Rats, Laboratory[Title/Abstract] OR Laboratory Rat[Title/Abstract] OR Laboratory Rats[Title/Abstract] OR Rat, Laboratory[Title/Abstract]))) OR (("Animal Experimentation"[Mesh]) OR (Experimentation, Animal[Title/Abstract] OR Animal Research[Title/Abstract] OR Research, Animal[Title/Abstract] OR Animal Experimental Use[Title/Abstract] OR Animal Experimental Uses[Title/Abstract] OR Experimental Use, Animal[Title/Abstract] OR Experimental Uses, Animal[Title/Abstract] OR Animal Experiments[Title/Abstract] OR Animal Experiment[Title/Abstract] OR Experiment, Animal[Title/Abstract] OR Experiments, Animal[Title/Abstract]))) AND (("Acupuncture Therapy"[Mesh]) OR (Acupuncture Treatment[Title/Abstract] OR Acupuncture Treatments[Title/Abstract] OR Treatment, Acupuncture[Title/Abstract] OR Therapy, Acupuncture[Title/Abstract] OR Pharmacoacupuncture Treatment[Title/Abstract] OR Treatment, Pharmacoacupuncture[Title/Abstract] OR Pharmacoacupuncture Therapy[Title/Abstract] OR Therapy, Pharmacoacupuncture[Title/Abstract] OR Acupotomy[Title/Abstract] OR Acupotomies[Title/Abstract]))) AND (("Depressive Disorder"[Mesh]) OR (Depressive Disorders[Title/Abstract] OR Disorder, Depressive[Title/Abstract] OR Disorders, Depressive[Title/Abstract] OR Neurosis, Depressive[Title/Abstract] OR Depressive Neuroses[Title/Abstract] OR Depressive Neurosis[Title/Abstract] OR Neuroses, Depressive[Title/Abstract] OR Depression, Endogenous[Title/Abstract] OR Depressions, Endogenous[Title/Abstract] OR Endogenous Depression[Title/Abstract] OR Endogenous Depressions[Title/Abstract] OR Depressive Syndrome[Title/Abstract] OR Depressive Syndromes[Title/Abstract] OR Syndrome, Depressive[Title/Abstract] OR Syndromes, Depressive[Title/Abstract] OR Depression, Neurotic[Title/Abstract] OR Depressions, Neurotic[Title/Abstract] OR Neurotic Depression[Title/Abstract] OR Neurotic Depressions[Title/Abstract] OR Melancholia[Title/Abstract] OR Melancholias[Title/Abstract] OR Unipolar Depression[Title/Abstract] OR Depression, Unipolar[Title/Abstract] OR Depressions, Unipolar[Title/Abstract] OR Unipolar Depressions[Title/Abstract]))

**Web of science**

1: (TS=(Rabbits)) OR TS=(Rabbit or New Zealand Rabbits or Rabbit, New Zealand or Rabbits, New Zealand or Zealand Rabbit, New or Zealand Rabbits, New or New Zealand White Rabbit or New Zealand Rabbit or New Zealand White Rabbits or NZW Rabbits or NZW Rabbit or Rabbit, NZW or Rabbits, NZW or Rabbit, Domestic or Domestic Rabbit or Domestic Rabbits or Rabbits, Domestic or Oryctolagus cuniculus or cuniculus, Oryctolagus or Belgian Hare or Hare, Belgian or Chinchilla Rabbits or Chinchilla Rabbit or Rabbit, Chinchilla or Rabbits, Chinchill)

2: (TS=(Mice)) OR AB=(Mus or Mouse or Mus domesticus or Mus musculus domesticus or domesticus, Mus musculus or Mus musculus or Mice, House or House Mice or Mouse, House or House Mouse or Mouse, Swiss or Swiss Mouse or Swiss Mice or Mice, Swiss or Mice, Laboratory or Laboratory Mice or Mouse, Laboratory or Laboratory Mouse)

3: (TS=(Rats)) OR AB=(Rat or Rattus or Rattus norvegicus or Rats, Norway or Rats, Laboratory or Laboratory Rat or Laboratory Rats or Rat, Laboratory)

4: (TS=(Animal Experimentation)) OR AB=(Experimentation, Animal or Animal Research or Research, Animal or Animal Experimental Use or Animal Experimental Uses or Experimental Use, Animal or Experimental Uses, Animal or Animal Experiments or Animal Experiment or Experiment, Animal or Experiments, Animal)

5: #1 OR #2 OR #3 OR #4

6: (TS=(Acupuncture Therapy)) OR AB=(Acupuncture Treatment or Acupuncture Treatments or Treatment, Acupuncture or Therapy, Acupuncture or Pharmacoacupuncture Treatment or Treatment, Pharmacoacupuncture or Pharmacoacupuncture Therapy or Therapy, Pharmacoacupuncture or Acupotomy or Acupotomies)

7: (TS=(Depressive Disorder)) OR AB=(Depressive Disorders or Disorder, Depressive or Disorders, Depressive or Neurosis, Depressive or Depressive Neuroses or Depressive Neurosis or Neuroses, Depressive or Depression, Endogenous or Depressions, Endogenous or Endogenous Depression or Endogenous Depressions or Depressive Syndrome or Depressive Syndromes or Syndrome, Depressive or Syndromes, Depressive or Depression, Neurotic or Depressions, Neurotic or Neurotic Depression or Neurotic Depressions or Melancholia or Melancholias or Unipolar Depression or Depression, Unipolar or Depressions, Unipolar or Unipolar Depressions)

8: #5 AND #6 AND #7

**The Cochrane Library**

#1 MeSH descriptor: [Acupuncture Therapy] explode all trees

#2 Acupuncture Treatment or Treatment, Acupuncture or Acupuncture Treatments or Therapy, Acupuncture or Pharmacoacupuncture Therapy or Pharmacoacupuncture Treatment or Therapy, Pharmacoacupuncture or Treatment, Pharmacoacupuncture or Acupotomies or Acupotomy

#3 #1 or #2

#4 MeSH descriptor: [Depressive Disorder] explode all trees

#5 Melancholias or Melancholia or Endogenous Depressions or Depressions, Endogenous or Endogenous Depression or Depression, Endogenous or Depressive Syndrome or Depressive Syndromes or Syndrome, Depressive or Syndromes, Depressive or Unipolar Depression or Depressions, Unipolar or Unipolar Depressions or Depression, Unipolar or Depressive Disorders or Neurosis, Depressive or Depressive Neuroses or Disorders, Depressive or Depressive Neurosis or Disorder, Depressive or Neuroses, Depressive or Neurotic Depressions or Depressions, Neurotic or Neurotic Depression or Depression, Neurotic

#6 #4 or #5

#7 MeSH descriptor: [Rabbits] explode all trees

#8 Domestic Rabbit or Oryctolagus cuniculus or Domestic Rabbits or cuniculus, Oryctolagus or Rabbit, Domestic or Rabbits, Domestic or Rabbit, Chinchilla or Chinchilla Rabbit or Rabbits, Chinchilla or Chinchilla Rabbits or Belgian Hare or Hare, Belgian or Rabbit or NZW Rabbits or Rabbit, New Zealand or Zealand Rabbits, New or Rabbits, NZW or Zealand Rabbit, New or Rabbit, NZW or New Zealand White Rabbit or Rabbits, New Zealand or New Zealand White Rabbits or NZW Rabbit or New Zealand Rabbits or New Zealand Rabbit

#9 #7 or #8

#10 MeSH descriptor: [Mice] explode all trees

#11 Mus domesticus or Mus musculus domesticus or domesticus, Mus musculus or Mice, Swiss or Swiss Mice or Mouse, Swiss or Swiss Mouse or Mus musculus or Mouse, House or House Mice or Mice, House or House Mouse or Mus or Mouse or Mouse, Laboratory or Mice, Laboratory or Laboratory Mouse or Laboratory Mice

#12 #10 or #11

#13 MeSH descriptor: [Rats] explode all trees

#14 Rats, Laboratory or Laboratory Rats or Laboratory Rat or Rat, Laboratory or Rattus or Rat or Rats, Norway or Rattus norvegicus

#15 #14 or #13

#16 MeSH descriptor: [Animal Experimentation] explode all trees

#17 Experiments, Animal; Animal Experiments; Animal Experiment; Experiment, Animal; Experimental Use, Animal; Experimental Uses, Animal; Animal Research; Animal Experimental Use; Experimentation, Animal; Research, Animal; Animal Experimental Uses

#18 #16 or #17

#19 #18 or #15 or #12 or #9

#20 #19 and #3 and #6

**Embase**

#20 #3 AND #6 AND #19 206

#19 #9 OR #12 OR #15 OR #18 5307726

#18 #16 OR #17 3014158

#17 'animal experimentation' OR 'animal physical conditioning' OR 'animal studies' OR 'animal study' OR 'animal trial' OR 'experiment, animal' OR 'physical conditioning, animal' 62202

#16 'animal experiment'/exp 2977187

#15 #13 OR #14 2057621

#14 'rats' OR 'rattus' 1120715

#13 'rat'/exp 1985955

#12 #10 OR #11 2294624

#11 'mice' OR 'mus (genus)' OR 'newborn mice' 1397346

#10 'mouse'/exp 2167989

#9 #7 OR #8 408244

#8 'leporid' OR 'leporids' OR 'rabbit' OR 'rabbits' OR 'rabbits and hares' 301973

#7 'leporidae'/exp 301551

#6 #4 OR #5 55885

#5 'acupuncture therapy' OR 'shonishin' 2378

#4 'acupuncture'/exp 55801

#3 #1 OR #2 620391

#2 'central depression' OR 'clinical depression' OR 'depressive disease' OR 'depressive disorder' OR 'depressive episode' OR 'depressive illness' OR 'depressive personality disorder' OR 'depressive state' OR 'depressive symptom' OR 'depressive syndrome' OR 'mental depression' OR 'parental depression' 75064

#1 'depression'/exp

# **Supplementary Figure 1**


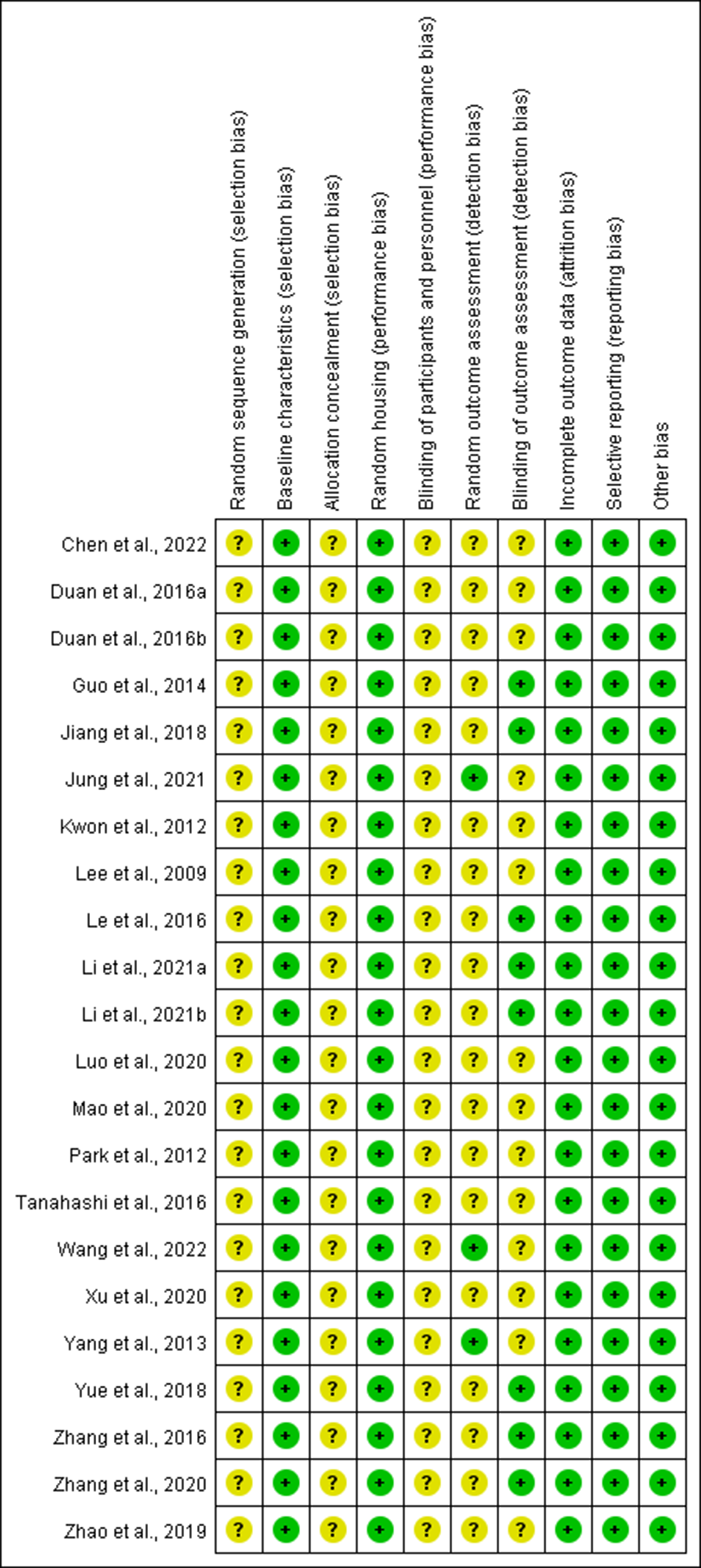


**Supplementary Figure 1.** Risk of bias assessment.

# **Supplementary Figure 2**


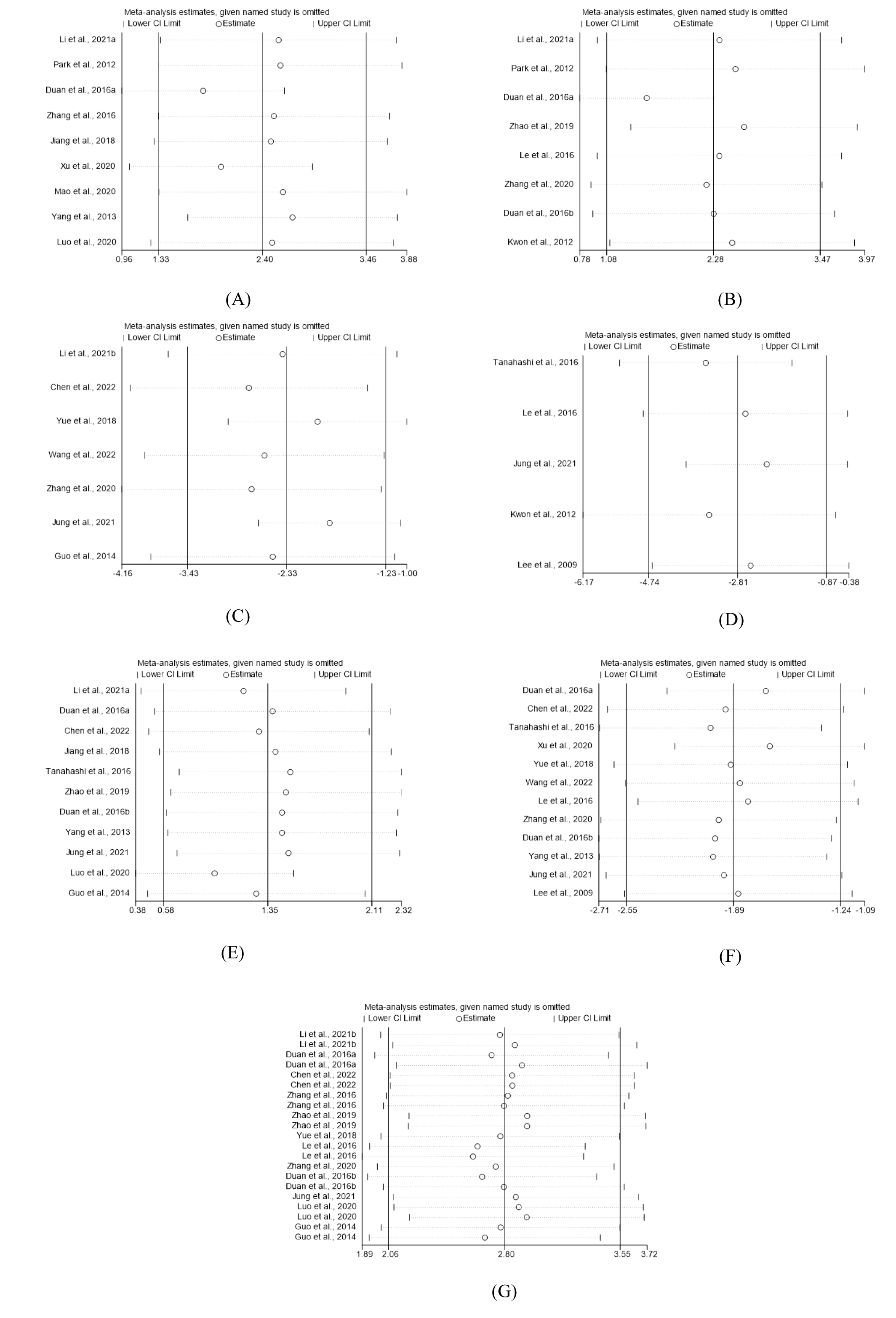


**Supplementary Figure 2.** The sensitivity analysis.

# **Supplementary Figure 3**


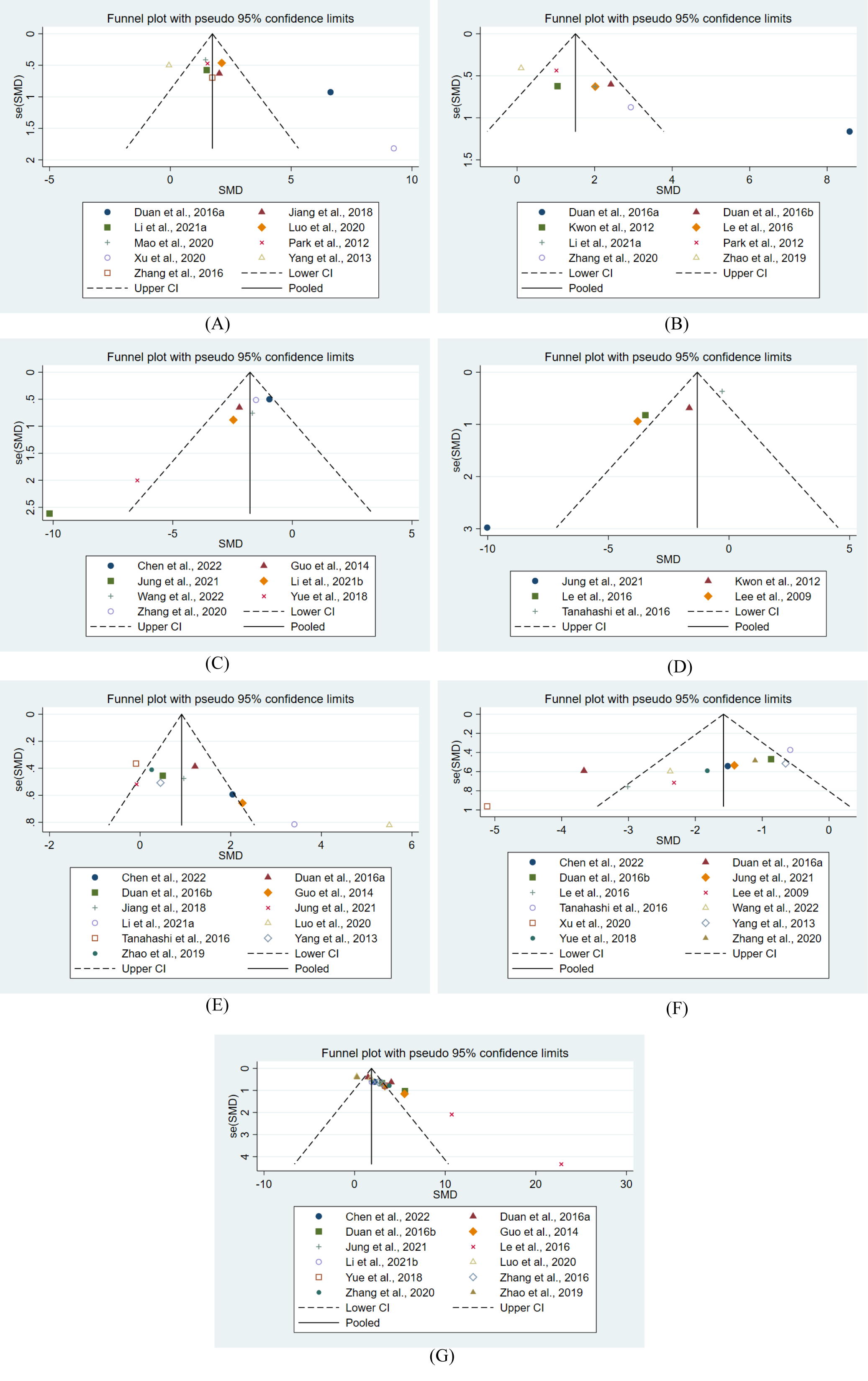


**Supplementary Figure 3.** The publication bias.
